# Well and Spring Cleaning

**Published:** 1859-03

**Authors:** 


### WELL AND SPRING CLEANING.

As spring is approaching, we earnestly advise all persons who use well water and spring water, to have both wells and springs thoroughly cleaned out, and then washed out in early May and also during October, as there is strong reason to believe that the settlings which have accumulated, including decayed vegetation, impart their disease engendering qualities to the water, and thus originate some of the most dangerous forms of low or typhoid fever, at a time of the year when the weather is so cool as to preclude the idea of their arising from vegetable decomposition. The stench of the *debris* at the bottom of wells should induce all cleanly persons to expurgate them thoroughly, aside from considerations of health.

